# Effectiveness and costs of an implemented primary HPV cervical screening programme in Sweden – A population based cohort study

**DOI:** 10.1016/j.pmedr.2021.101675

**Published:** 2021-12-23

**Authors:** Lovisa Bergengren, Linda Ryen, Clelia Flodström, Helena Fadl, Ruzan Udumyen, Mats G. Karlsson, Gisela Helenius

**Affiliations:** a Department of Obstetrics and Gynaecology, Faculty of Medicine and Health, Örebro University, Örebro, Sweden; b Department of Women’s Health, Örebro University Hospital, Örebro, Sweden; c Clinical Epidemiology and Biostatistics, School of Medical Sciences, Örebro University, Örebro, Sweden; d University Health Care Research Centre, Faculty of Medicine and Health, Örebro University, Örebro, Sweden; e Department of Laboratory Medicine, Faculty of Medicine and Health, Örebro University, Örebro, Sweden

**Keywords:** Cervical cancer, Screening, Human papilloma virus (HPV), Health economy

## Abstract

Swedish guidelines recommend cervical screening with primary HPV for women ≥ 30 years of age. The aim of this study was to compare an implemented HPV cervical screening programme in the Region of Örebro County from September 1, 2016, with the former cytology-based screening programme.

The clinical effectiveness by means of number of high-grade squamous intraepithelial lesions (HSILs) and cervical cancer cases detected in histology within 12 months after the screening test, together with cost implications were the main outcomes. Data were retrieved from the Swedish National Cervical Screening Registry between the years 2014–2015 (cytology based screening) and 2017–2018(HPV based screening), including screening information such as invitations and cytology and histology diagnoses.

The detection rate of HSIL + among women ≥ 30 years of age was 1.2 times higher with HPV screening, but data revealed an increase in direct colposcopy referral rate by 54% and a higher percentage of irrelevant findings (≤LSIL). Screening based on HPV for women ≥ 30 has increased yearly cost from 1 to 1.3 million EUR, while increasing the number of HSIL + identified. Two thirds of the total costs are from visits for screening samples in the programme.

HPV screening detected more cases of HSIL + compared to cytology screening among women ≥ 30 although high colposcopy rate, high rate of clinical irrelevant findings and higher costs were shown in the HPV-based screening programme, which implies that alterations in the screening programme in the future are important to consider.

## Highlights

• In an HPV-based cervical screening programme more HSIL + cases was detected but the clinical effectiveness comes with higher rates of colposcopy and clinical irrelevant findings.
• In an HPV-based cervical screening programme two thirds of the cost, come from screening visits with sampling.
• From a health economic perspective, our data emphasize the need for considering alterations in the screening programme including more specific triage analysis and the implementation of self-sampling.

## 1 Introduction

**Fig. 1:**
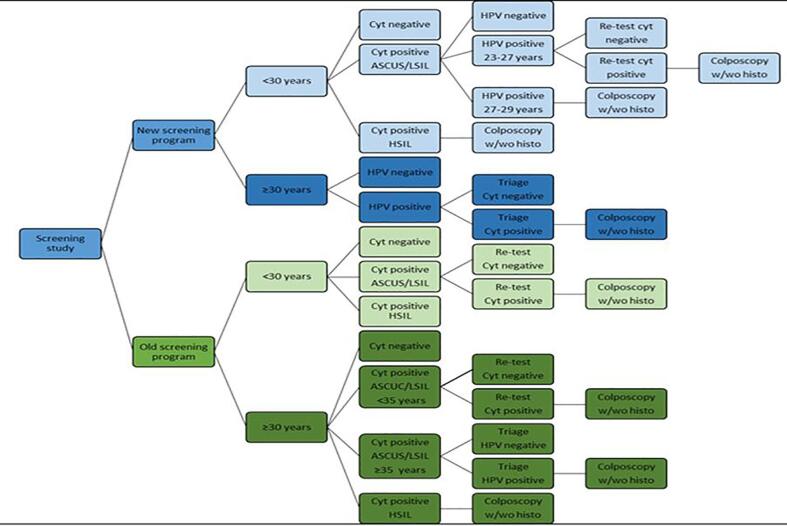
Flowchart illustrating the old and the new screening programmes.

Cervical screening programmes for detection of precursor lesions to invasive cervical cancer have been ongoing in several countries for decades and have been successful in prevention of cervical cancer (Gustafsson et al., 1997, Vaccarella et al., 2014). The recognition of persistent high-risk human papillomavirus (hrHPV) infection as the major cause of cervical cancer (zur Hausen H., 2000) has led to new screening recommendations by the European Union (EU) (Arbyn et al., 2010, Anttila et al., 2015) and the World Health Organization (WHO) (World Health Organization, 2014). Shifting from primary screening with cytology in favour to primary human papillomavirus (HPV) screening is completed or in progress in several countries (Aitken et al., 2019); and data support the evidence of higher detection rate of high-grade squamous intraepithelial lesions (HSIL) and cancer, HSIL+, with this strategy (Aitken et al., 2019, Rebolj et al., 2019). In Sweden, an HPV primary screening policy was issued in 2015 by the National Board of Health and Welfare (Board, 2015). Screening with HPV is recommended between the ages of 30 and 70, with sampling every three to seven years (Fig. 1), while cytology-based screening is still favoured for women 23–29 years of age due to high prevalence of transient hrHPV infections (Ho et al., 1998, Monsonego et al., 2015, Wheeler et al., 2013).

Consistent evidence from studies indicate that HPV-based screening is more sensitive and detects more HSIL and has increased efficiency in cancer prevention compared to cytology-based screening, but still has a lower specificity (Arbyn et al., 2012, Ronco et al., 2014, Wright et al., 2015). Therefore, the screening strategy with primary HPV has been proposed to increase the number of clinical examinations following a positive screening test, which reports from implemented programmes also confirm (Aitken et al., 2019, Rebolj et al., 2019). Depending on age, cytology and HPV genotyping as triage or re-tests of positive samples are recommended in order to increase the specificity for identifying the women who are at higher risk of cancer development and in greatest need for a follow-up. In the old screening programme, HPV was used as triage on aberrant cytology samples, whereas in the present (new) programme the triage method is cytology if HPV test is positive.

The cost-effectiveness of implemented HPV screening programmes is not well explored and most data is on models and trials (Bistoletti et al., 2008, van Rosmalen et al., 2012, Mezei et al., 2017). High-quality studies are lacking regarding real life evaluation of clinical effectiveness combined with health care resource use for implemented HPV primary screening programmes compared to cytology-based screening programmes. Thus, the aim of this study was to evaluate the actual effectiveness and cost implications of an implemented HPV screening programme in a real setting compared with the former cytology-based screening programme.

## 2 Materials and methods

### 2.1 Study population

The study population included all women residing in the Region of Örebro County, Sweden, who were invited to the organized cervical screening programme according to national policies and had their screening samples taken between the years 2014–2015(cytology based screening) and 2017–2018(HPV based screening). All women deregistered from the screening due to prior hysterectomy or own request or with a history of invasive carcinoma at inclusion were excluded. On September 1, 2016, this region shifted the screening algorithm from cytology as primary screening method to primary HPV screening. However, cytology-based screening continued among the women < 30 years of age (Fig. 1).

The old screening programme included women 23 to 59 years of age, plus women with prolonged screening after 59 due to prior histologically verified HSIL in excision material, who attended screening between January 1, 2014 and December 31, 2015, following invitation. The new screening programme included women aged between 23 and 70 who attended screening between January 1, 2017 and December 31, 2018, following invitation. The maximum time allowed between invitation date and sample date equals 1.5 years (=548 days) meaning that invitations were sent out before the sampling-period for some. Since the major changes in the screening programme were introduced to women ≥ 30, the study population is presented and discussed in two age groups: younger or older than 30 years of age (Fig. 1).

### 2.2 Data sources

In Sweden, all women 23–70 years of age are offered screening, and the screening programme is publically funded. Data on all cervical cytology and histopathology on women born between 1948 and 1992 for the old screening programme and for women born between 1948 and 1995 for the new screening programme were retrieved from the Swedish National Cervical Screening Registry (NKCx) ((NKCx) TSNCSR, 2020). Additionally, the local laboratory information management system (Flexlab/SymPathy, Tieto, Sweden) were used for cytology and histology follow-up data for 2019 for women invited to screening during 2018 since the NKCx registry by that time only was updated to 2018.

For cost estimations, unit costs were collected from regional administrative sources: the Cost per Patient database for clinical examinations, laboratory tariffs for triage analysis and negotiated prices for screening visits.

### 2.3 Description of cytology, HPV testing and histology

Screening samples (primary samples and retests) were collected by midwives at health centres in both screening programmes. Cytology slides were prepared from liquid- based samples (ThinPrep, Hologic, Marlborough, MA, USA) and assessed by IAC certified cytotechnicians who classified findings according to present guidelines with atypical squamous cells of undetermined significance (ASCUS), atypical squamous cells, cannot exclude high grade lesion (ASC-H), low-grade squamous intraepithelial lesion (LSIL), high-grade squamous intraepithelial lesion (HSIL), squamous cell carcinoma, atypical glandular cells (AGC), adenocarcinoma in situ (AIS) or adenocarcinoma (Arbyn et al., 2010).

For HPV-testing, samples were analysed with Aptima HPV assay (Hologic, Marlborough, MA, USA) that detect 14 high risk HPV genotypes 16, 18, 31, 33, 35, 39, 45, 51, 52, 56, 58, 59, 66 and 68.

In the local hospitals, gynaecologists carried out all follow-up colposcopies and histological sampling. Concerning histopathology, cervical punch and excision biopsies were formalin fixed, paraffin embedded and thereafter slides were cut at 4 μm, stained with hematoxylin and eosin and evaluated by pathologists according to present WHO classification (Kurman, 2014). The presence or absence of the transformation zone and the presence of dysplasia in the surgical margins were noted (Reich et al., 2017).

### 2.4 Outcomes

The main outcome is HSIL or invasive cervical cancer (HSIL + ) identified at histopathological examination within 12 months after the screening test. Women could have several histopathological examinations within 12 months after cervical cytology, in which case, the highest-ranked diagnosis was used as the outcome measure. The study also aimed to estimate costs and compare those between the two screening strategies, both in relation to number of women screened and number of histological HSIL + identified. This includes looking at number of follow-up visits stipulated by each programme. Follow-up visits of interest included cervical cell samples, colposcopy and/or histopathological investigations and treatments with cervical punch biopsies or cervical cone biopsies.

### 2.5 Estimation of costs

Costs per programme were estimated from a health care perspective, based on the actual frequencies of screening tests, triage analyses and follow-up examinations and treatments performed within each programme, multiplied by the 2018 unit costs. The cost types included as well as the unit costs used for valuation of resource use are presented in the top row in table 2a and b. Costs are presented in Euros based on the average 2018 exchange rate (1 EUR = 10.26 SEK).

Estimated costs are presented as average yearly cost, cost per 1000 women screened and cost per histological HSIL + identified for each programme respectively.

### 2.6 Statistics

Data are shown in absolute numbers and as percentages. Clinical outcomes are presented for the whole study period and concerning health costs per year. Comparison of the old and the new screening programme for women ≥ 30 years of age, based on numbers of HSIL + detected at histology, was done by Pearson’s chi square test. STATA 14/SE and SPSS were used for statistical analysis.

### 2.7 Ethical approval

This study was approved by the regional ethics committee in Uppsala (Dnr 2017/297). The approval gave consent to get screening data from NKCx concerning women who were invited and participated in cervical screening between the years 2014–2015 and 2017–2018, as well as data from local registers for the same period. All data were analysed anonymously by the researchers.

## 3 Results

In total, 27,362 women aged 23–66 years attended screening between January 1, 2014 and December 31, 2015, following an invitation within the old programme, while 8,483 women did not attend. That resulted in total 27,438 screening samples. In comparison, 33,026 women aged 23–70 years attended screening between January 1, 2017 and December 31, 2018, following an invitation within the new screening programme while 8,422 women did not attend. Overall, 33,088 screening samples were taken at a first visit. The participation rate in the new screening programme was 80% and in the old screening programme 76%.

**Supplementary figure 1:**
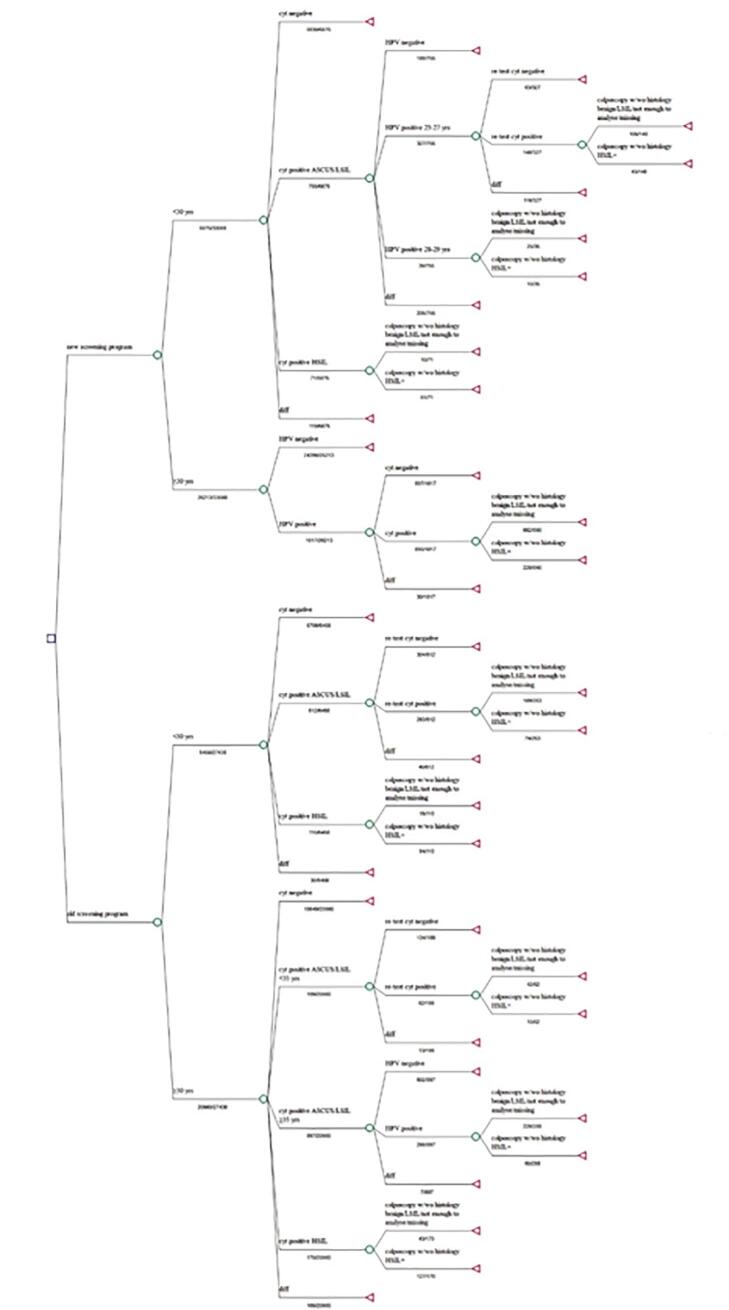

Yearly, 13,719 samples were taken in the old programme and 16,544 in the new programme. In Table 1, the overall results of the screening programmes are presented by age. In supplemental Fig. 1, detailed numbers for the programmes are presented.

### 3.1 Women < 30 years of age

In the old screening programme, 94% of all screening samples among women < 30 years of age had an outcome with normal cytology and/or histology, compared with 90% in the new programme. Of the punch and cervical excision biopsies, 39.6% were identified with HSIL + as the highest histopathological endpoint in the old programme compared with 39.4% in the new (Table 1). In this age group, the old programme detected seven cervical cancers compared to the new programme that detected one case, a detection rate of cervical cancer of 1.1/1000 women in the old programme vs 0.17 /1000 in the new programme.

**Table 1:** Descriptive numbers of the old and new screening programmes in the Region of Örebro County.

	Women screened n	Cytology Samples n	HPV samples n	Direct colpo-scopy n (%)	Total colpo-scopy n (%)	Cervical punch biopsy n	Cervical cone biopsy n	Histology n		
								Benign	LSIL	HSIL+
Old programme*										
23–29	6439	6458	–	110 (1.7)	373 (5.8)	255	169	66	46	168
30–39	7501	7518	204	182 (2.4)	234 (3.1)	146	135	39	37	107
40–49	8735	8756	446	196 (2.2)	196 (2.2)	104	91	41	30	67
50–59	4657	4671	236	78 (1.7)	78 (1.7)	44	40	18	16	22
60–70	33	35	4	2 (5.7)	2 (5.7)	0	1	0	0	1
Total	27,362	27,438	890							
New programme**										
23–29	6826	6875	550	106 (1.5)	254 (3.7)	212	77	31	50	114
30–39	7916	812	7921	430 (5.4)	430 (5.4)	310	132	66	110	135
40–49	8312	519	8316	264 (3.2)	264 (3.2)	177	59	44	80	60
50–59	5359	304	5362	128 (2.9)	128 (2.9)	75	21	26	29	19
60–70	4613	182	4614	68 (1.5)	68 (1.5)	44	15	9	22	14
Total	33,026	8692	26,763							

*time period between January 2, 2014 and December 30, 2015

**time period January 5, 2017 and December 28, 2018

### 3.2 Women ≥ 30 years of age

Overall, 97.2 % of the screening samples in the old programme had an outcome with normal cytology and/or histology, compared with 97% in the new programme. Samples positive for hrHPV in the new programme was 6.9%, which equals the HPV prevalence among the 26,200 women primarily tested for hrHPV. These samples were analysed for cytology as a triage and 49% were identified with aberrant cytology with ASCUS or above, leading to colposcopy. Comparable results in the old programme were 6.8% positive samples in cytology (ASCUS + ). After possible triage test or retest, a total of 35.5% of these led to colposcopy.

Direct colposcopy rate, that is, positive samples in the screening programme resulting in colposcopy without further retest, were 1.9 times higher per year in the new programme in this age group. This led to a detection of HSIL + in 25.5% of these examinations in the new programme, whereas the corresponding detection rate in the old programme was 43%. The number of colposcopies was 229 and 445 per year in the old and new programme respectively, with corresponding numbers of cervical cone biopsies of 134 and 114 per year in the old and new programme respectively (Table 1, Table 2a, Table 2b).

As endpoint, the highest ranked histopathological sample within 12 months after screening-test was used. Histology samples classified as benign or LSIL were seen in 48% of the endpoints in the old programme, compared to in 63% in the new. In total over the study period among women aged ≥ 30 years, the detection rate of cancer in the screening was 0.29/1000 (n = 6) women for the old programme and 0.40/1000 women (n = 11) for the new. During the study period 191 and 217 HSIL were detected in the old vs the new programme. When comparing the whole group of women ≥ 30 years of age, there was no statistically significant difference in detection of HSIL + between the old and the new programmes (P = 0.429). Although when comparing women 35–59 years of age, the age where there are women in both programmes that have been tested for both HPV and cytological abnormalities, but where the order of tests has been switched, the old programme detected significantly more HSIL+ (P = 0.005) if screening showed ASCUS/LSIL and HPV positivity. When also including HSIL cytology, the old programme also detected significantly more HSIL+ (P = < 0.001) (Supplemental Table 1).

### 3.3 Costs

The average yearly cost of the new programme, including also women < 30, amounted to EUR 1.6 million in 2018 price level, to be compared with the corresponding yearly cost of EUR 1.4 million for the old programme. For both programmes, screening visits represent nearly two thirds of total costs. The cost per 1000 women is approximately EUR 100,000 in both programmes. The average cost per HSIL + identified is about EUR 9,600 in the new and EUR 7,600 in the old programme.

Comparing the programmes in terms of both average yearly costs and effectiveness shows that the new programme inferred higher costs but a lower number of HSIL + were identified.

### 3.4 Women < 30 years of age

For women under 30 years (Table 2a), the yearly cost for the new programme is lower than the corresponding cost for the old programme (EUR 362,000 compared to EUR 415,000), but the cost per 1000 women is comparable between the programmes. On the other hand, the introduction of triage analysis in the new programme adds to the costs compared to the old programme. The average cost per HSIL + identified is EUR 6,335 in the new programme and EUR 4,971 in the old programme.

**Table 2a:** Estimations of costs for old* and new** screening programmes in the Region of Örebro County for women age < 30 between January 2014 to December 2015 and January 2017 to December 2018. Costs are presented as average costs in EUR *** per year and per 1000 women in 2018 price level.

	Total cost	Screening visits (including re-tests)	Triage analysis	Colposcopy	Cervical punch biopsy	Cervical excision biopsy	Cellscrape
*Unit cost (EUR 2018)*		*66*	*37*	*403*	*416*	*648*	*1,246*
Cost per year, old program*	415,397	230,598	–	75,091	53,063	54,777	1,869
Cost per year, new program**	362,340	232,401	10,078	48,920	44,115	24,958	1,869
Cost per 1,000 women, old program*	129,025	71,625	–	23,324	16,482	17,014	580
Cost per 1,000 women, new program**	124,387	79,781	3,460	16,794	15,144	8,568	641

*time period between January 2, 2014 and December 30, 2015

**time period January 5, 2017 and December 28, 2018

*** 1 Euro = 10.26 SEK (average exchange rate 2018)

Comparing the programmes in terms of both average yearly costs and effectiveness shows that the new programme inferred lower costs but a lower number of HSIL + were identified.

### 3.5 Women ≥ 30 years of age

For women 30 years and above (Table 2b), yearly cost of the new programme is 32% higher than that of the old programme (EUR 1.3 million compared to EUR 1 million). In addition, the cost per 1000 women is higher in the new programme, even if the cost per 1000 women for screening visits is higher in the old programme. The average cost per HSIL + identified is EUR 11,200 for the new programme and EUR 9,800 for the old programme.

**Table 2b:** Estimations of costs for old* and new** screening programmes in the Region of Örebro County for women age ≥ 30 between January 2014 to December 2015 and January 2017 to December 2018. Costs are presented as average costs in EUR *** per year and per 1000 women in 2018 price level.

	Total cost	Screening visits (including re-tests)	Triage analysis	Colposcopy	Cervical punch biopsy	Cervical excision biopsy	Cell scrape
*Unit cost (EUR 2018)*		*66*	*373*	*403*	*416*	*648*	*1,246*
Cost per year, old program*	969,081	694,285	16,308	102,671	61,178	86,541	8,097
Cost per year, new program**	1,280,028	859,715	32,744	179,171	126,102	73,576	8,720
Cost per 1,000 women, old program*	92,620	66,356	1,559	9,813	5,847	8,271	774
Cost per 1,000 women, new program**	97,712	65,627	2,500	13,677	9,626	5,616	666

*time period between January 2, 2014 and December 30, 2015

**time period January 5, 2017 and December 28, 2018

*** 1 Euro = 10.26 SEK (average exchange rate 2018).

Comparing the programmes in terms of both average yearly costs and effectiveness shows that the new programme inferred higher costs but also increased the number of HSIL + identified. The incremental cost of identifying one additional HSIL + in the new programme compared to the old amounts to EUR 20,760.

## 4 Discussion

Internationally, HPV test is recommended as the test of choice for cervical screening (Anttila et al., 2015). This is based on the high negative predictive value of HPV tests, the lower cumulative incidence of HSIL in test negative women with HPV compared with cytology, and the evidence of more efficacious prevention against cervical cancer (Ronco et al., 2014). The Region of Örebro County implemented HPV as primary screening September 1, 2016 and the purpose of this study was to evaluate the screening programmes’ clinical effectiveness and costs. To the best of our knowledge, this report is the first in highlighting both clinical effectiveness and costs from a real setting and current organization, so called real world data (Makady et al., 2017), of an implemented screening programme with an hrHPV mRNA method.

The participation rate increased from 76% to 80% when switching screening programme. This together with inclusion of women up to 70 years of age in the new screening programme, increased the total number of women screened with 20%. Naturally, this increased total costs for the screening programme. Although this is expected, it is relevant for decision makers due to budget impact but also as an input for possible discussions on screening intervals and cost effectiveness.

Prevalence of hrHPV in our data for women ≥ 30 years of age was 6.9%, which is comparable with data from another region in Sweden (7%) (Lindroth et al., 2019) and a Dutch screening study (7.5%) when using the same HPV method as in this study (Huijsmans et al., 2016). In this study, as well as in other studies with this study design, it is not possible to know if negative samples were classified falsely as normal, since no histological examination is performed on women with negative test results.

Overall, the old programme shows higher detection of HSIL + cases compared to the new programme when including all women 23–70 years of age. The results differ between the women < 30 where HPV primary screening has not been introduced, and women ≥ 30 years where HPV is implemented. Data show a higher detection rate of HSIL + cases in the old programme among the younger age group while the new programme identifies more HSIL + cases for women 30 and above. Among the women that were screened for the first time during 2017–2018, approximately 60% was vaccinated (Lei et al., 2020). That can explain the lower number of HSIL + cases found in the new programme in women < 30. Although, since the individual vaccination status is unknown in this study and the fact that women in the young age groups in 2014–2015 also were vaccinated to some extent(25–30%), this study cannot get any closer to explaining the difference in results among women < 30.

Among women ≥ 30 years of age who were screened for HPV in the new screening programme, the detection rate of HSIL + was almost 1.2 times higher than in the old (114vs99cases). This is in line with other studies where detection for HSIL + with HPV screening increased by 1.3 and 1.4 times (Aitken et al., 2019, Rebolj et al., 2019). Concerning detection rates of HSIL + per 1000 women among those aged ≥ 30 years, the new programme finds less cases with 8.7 /1000 women compared to 9.4 /1000 women in the old programme, which does not align with the Dutch study (Aitken et al., 2019) that increased the detection rate per 1000 women as well. Here it is important to emphasize that women in Sweden are screened with a 3-year interval between the ages 30–50 and thus the women can be considered more well screened than in a screening programme with a 5-year interval. In addition, this renders many more women to screening.

For women aged ≥ 30 years, 48% of the histology samples are considered clinical irrelevant findings (≤LSIL) in the old programme and 63% in the new programme. The difference with 1.3 times more irrelevant findings in the new programme (≥30) is lower compared to Aitken et al. who report 2.2 times more irrelevant findings with primary HPV screening (Aitken et al., 2019). This could be a result of a higher specificity in an mRNA-based HPV-test, used here, than DNA-based methods (Arbyn et al., 2015, Haedicke and Iftner, 2016). Altogether, this indicate the need for a triage method with higher specificity.

Our data with an increase in direct colposcopy referral rate of 54% in the new screening programme (≥30) is exactly in line with a report by Lindroth et al (Lindroth et al., 2019). One limitation in our study is however that the local registers could not give adequate information on how many colposcopies that had been carried out. Estimations on total number of colposcopies were done based on the cytology and histology results with the highest-ranked diagnosis. A limitation is that women could have several histopathological examinations within 12 months after cervical cytology, resulting in missed colposcopies in the calculation, as well as some colposcopies are missed that are not followed by histological sampling. Thus, this data underestimate the number of colposcopies, but for both the old and the new programme.

Another important observation is the difference of effectiveness between the study periods among women under the age of 30, despite that both programmes were cytology-based with just minor changes in the new programme. The direct colposcopy rate was nearly the same between the programmes, 1.5 % in the new programme versus 1.7 % in the old. However, in the old programme the total number of colposcopies was 1.5 times higher with 1.5 times more HSIL + cases detected. If the lower number of detected HSIL in women < 30 partly is also due to a true decrease of HSIL this could be a result of that a small part of this population was vaccinated. Unfortunately, we lack vaccination data on the individual level and therefore cannot analyse it further. In this study period, seven cancers were found in the old screening programme and only one in the new programme. A longer study period, or a larger study population, would be necessary for further analyses regarding this.

## 5 Conclusion

In the group where the alterations to HPV-screening is implemented, women aged ≥ 30 years; more HSIL + cases are detected. Even so, when including the whole screening population, higher colposcopy rates are seen, a higher rate of clinical irrelevant findings (≤LSIL), a lower rate of detected HSIL + cases per 1000 screened women and higher costs in the new screening programme, making alterations in the screening programme in the future is important to consider. Several studies have shown that longer screening intervals are safe for test negative women (Dillner, 2013, Dijkstra et al., 0000, Kitchener et al., 2011). If the programme would allow longer screening intervals for test negative women and utilize the full potential of the tests, the cost-effectiveness in the long term can be improved. We also conclude in our cost estimations that two thirds of the cost for the new programme comes from sampling (screening visits) and not from follow-up investigations and treatments. From a health economic perspective, this further emphasizes the future screening programmes to consider making alterations in the screening programme not just among women below 30 but also regarding the possibility of self-sampling and new triage methods.

## Funding

This article was funded by 10.13039/501100000008Region Örebro County Research Committee, grant OLL-841131.

### CRediT authorship contribution statement

**Lovisa Bergengren:** Writing, Conceptualization, Formal analysis, Methodology. **Linda Ryen:** Formal analysis, Methodology, Writing – review & editing, Data curation. **Clelia Flodström:** Writing, Formal analysis. **Helena Fadl:** Methodology, Formal analysis, Writing – review & editing. **Ruzan Udumyen:** Data curation, Formal analysis, Methodology, Writing – review & editing. **Mats G. Karlsson:** Supervision, Writing – review & editing. **Gisela Helenius:** Methodology, Formal analysis, Supervision, Writing – review & editing.

## Declaration of Competing Interest

The authors declare that they have no known competing financial interests or personal relationships that could have appeared to influence the work reported in this paper.

