# Supplementary material for: Effectiveness and costs of an implemented primary HPV cervical screening programme in Sweden – A population based cohort study
Source: Prev Med Rep. 2021 Dec 23;25:101675. doi: 10.1016/j.pmedr.2021.101675 (PMC8800063; doi:10.1016/j.pmedr.2021.101675)
Supplement: Supplementary data 1 [file mmc1.docx]

Supplemental Table 1

|  | No HSILplus | HSILplus | P value |
| --- | --- | --- | --- |
| Program |  |  |  |
| Old (age ≥ 30) | 20783 | 197 | 0.429 |
| New (age ≥ 30) | 25985 | 228 |  |
|  |  |  |  |
| Old *(ASCUS/LSIL, HPV+, age 35-59)* | 227 (79.4) | 59 (20.6) | 0.005 |
| New *(HPV+, ASCUS/LSIL, age 35-59)* | 399 (87.1) | 59 (12.9) |  |
|  |  |  |  |
| Old *(ASCUS/LSIL or HSIL, HPV+, age 35-59)* | 261 (65.1) | 140 (34.9) | <0.001 |
| New *(HPV+, ASCUS/LSIL or HSIL, age 35-59)* | 426 (76.1) | 134 (23.9) |  |
